# Strategic insights into pharmacogenomics coverage: a theory-informed SWOT analysis of UAE insurance stakeholders’ perspectives

**DOI:** 10.1186/s40246-025-00896-6

**Published:** 2025-12-29

**Authors:** Maram O. Abbas, Azhar T. Rahma, Iffat Elbarazi, Bassam R. Ali, George P. Patrinos, Hana Ghadibah, Fatma Al-Maskari

**Affiliations:** 1 Institute of Public Health, College of Medicine & Health Sciences, United Arab Emirates University, Al Ain, UAE; 2 Department of Genetics and Genomics, College of Medicine and Health Sciences , United Arab Emirates University , Al Ain, UAE; 3 Zayed Center for Health Sciences , United Arab Emirates University , Al Ain, UAE; 4 Department of Pharmacy School of Health Sciences , University of Patras , Patras, Greece; 5 Dubai Pharmacy College , Dubai, UAE

**Keywords:** Pharmacogenomics, Personalised medicine, Health policy, Insurance coverage, Healthcare implementation, UAE Healthcare System

## Abstract

**Background:**

Pharmacogenomic (PGx) testing improves treatment outcomes by tailoring therapy to a patient’s genetic profile. However, PGx implementation faces global challenges, including costs, reimbursement, and regulations. Initial PGx guidelines exist in the United Arab Emirates (UAE), but insurers’ perspectives remain understudied. This study explores insurers’ views on policies and strategies to expand PGx adoption and overcome implementation barriers.

**Methods:**

This qualitative study used a semi-structured interview design to explore the perspectives of twelve executive and middle management insurance representatives selected through purposive convenience and snowball sampling. Thematic analysis was conducted inductively, supported by comparative analysis, the Institutional Theory, the TAM, and SWOT analysis to interpret the findings.

**Results:**

Analysis revealed variable awareness of PGx, highlighting both perceived benefits and significant barriers. Key findings included economic constraints, limited physician and public awareness, and policy challenges related to cost-effectiveness and infrastructure. Ethical and privacy concerns were minimal but were noted, with potential implications for insurance premiums. Participants stressed the need for collaborative efforts to align PGx with UAE healthcare goals and highlighted the role of advanced health information systems in facilitating integration. Differences emerged between executive and middle-level management: the former emphasised strategic policies and long-term returns on investment, while the latter focused on practical operational barriers.

**Conclusions:**

Advancing PGx in the UAE requires local cost-effectiveness studies, clear government-led coverage guidelines, and collaborative action among insurers, providers, regulators, and academia. These findings may inform health systems with similar public–private insurance arrangements, where phased adoption strategies and education initiatives are key to sustainable implementation.

**Supplementary Information:**

The online version contains supplementary material available at 10.1186/s40246-025-00896-6.

## Introduction

Pharmacogenomics (PGx) is an innovative, rapidly advancing field that combines genetics and pharmacology and has the potential to transform modern healthcare [1]. By tailoring treatments to individual genetic profiles, PGx enhances therapeutic effectiveness, reduces adverse drug reactions, and provides a safer and more cost-efficient alternative to traditional “trial-and-error” prescribing [2–4].

Internationally, PGx implementation has evolved from isolated academic initiatives toward more structured, system-level integration within healthcare systems [5, 6]. High-income countries increasingly incorporate PGx into routine clinical decision-making, supported by digital health infrastructure, national biobanks, and precision medicine programmes. Examples include the Clinical Pharmacogenetics Implementation Consortium (CPIC) in the USA, which provides guidelines for more than 300 drug–gene interactions [7], and the Dutch Pharmacogenetics Working Group (DPWG), which integrates recommendations into the national prescribing system [8]. Canada’s CPNDS has developed paediatric guidelines and pharmacovigilance systems [9], while European initiatives such as the U-PGx project have demonstrated the feasibility of preemptive testing across diverse health systems [10].

Despite these advances, global adoption of PGx remains inconsistent because of high implementation costs, regulatory gaps, limited physician awareness, and infrastructural constraints [11, 12]. Further challenges include variable evidence standards, inconsistent testing protocols, and limited integration of PGx results into electronic health records, all of which require coordinated engagement from patients, clinicians, payers, regulators, laboratories, and academic institutions [13–16].

In the United Arab Emirates (UAE), efforts to integrate PGx are emerging. Abu Dhabi’s Department of Health has issued PGx guidelines supporting personalised prescribing [17]. However, broader implementation remains limited. Prior UAE research, including Rahma et al. (2021), has highlighted systemic barriers such as stakeholder misalignment and regulatory gaps, offering valuable insight into the landscape of genomic medicine adoption [18]. Nevertheless, these studies did not examine the perspectives of specific stakeholder groups in depth.

Among these stakeholders, insurers play a particularly influential role, as they shape access, reimbursement, and the long-term viability of emerging technologies such as PGx in health systems with substantial private-sector participation, such as the UAE [19–21] Despite this influence, their perspectives remain largely unexplored, leaving a critical gap in understanding how PGx can be realistically integrated into the UAE’s healthcare systems.

Accordingly, this study investigates how insurers in the UAE evaluate the feasibility, cost-effectiveness, and operational implications of PGx testing, and how their perspectives intersect with those of regulators and healthcare providers to shape the broader implementation landscape.The study’s insights will help guide PGx implementation strategies in the UAE and other health systems with comparable insurance structures.

## Method

### Study design and setting

This qualitative study employed a semi-structured interview design to explore the perspectives of insurance sector representatives on the implementation of PGx testing within the UAE healthcare system. Data collection and reporting adhered to the Consolidated Criteria for Reporting Qualitative Research (COREQ) checklist (Table S1, Supplementary).

The study was conducted in the UAE, where ongoing healthcare reforms emphasise quality improvement and clinical innovation through national strategies and regulatory initiatives [22]. The UAE operates a mandatory health insurance model regulated by governmental health authorities and delivered through a combination of public and private healthcare providers. Insurers play a central operational role in evaluating service coverage, prior authorisation processes, and the cost-effectiveness and regulatory suitability of new medical technologies, including PGx [23]. Healthcare organisations in the UAE also adhere to international accreditation and performance frameworks, such as Joint Commission International (JCI) standards and Key Performance Indicators (KPIs), which structure clinical governance and service quality expectations.

The UAE’s highly diverse population, comprising a majority of expatriates from varied ethnic backgrounds, adds additional complexity to the integration of genomic medicine [24]. Although the UAE has outlined national priorities for advancing healthcare innovation, personalised medicine approaches such as PGx are still in early stages of implementation, positioning insurers as key operational stakeholders within this evolving system.

### Study tool: development and validation

A semi-structured interview guide was developed to support systematic exploration of key issues related to PGx within the insurance sector. A review informed the guide of the literature on PGx implementation, payer decision-making, and cost-effectiveness assessment frameworks [18, 25, 26]. To ensure contextual relevance, the draft guide was reviewed by academic researchers and insurance professionals with expertise in genomics, reimbursement, and healthcare policy, and subsequently refined based on their feedback.

The final guide covered five core areas: participants’ demographics and professional backgrounds; their general understanding of PGx; their perceptions of PGx testing; financial considerations and cost-effectiveness; and views on integration and system-level recommendations. The semi-structured format ensured consistency across interviews while allowing flexibility to explore unanticipated insights raised by participants.

### Sample size and sampling

A purposive sampling strategy was primarily used to recruit participants actively involved in insurance decision-making regarding healthcare coverage in the UAE. To ensure the selection of relevant and informed participants, the research team began by preparing a list of insurance companies based on publicly available information from the official website of the UAE Insurance Association. Companies were categorised according to market share, size, and type of operations, distinguishing between full-service insurance providers and third-party administrators (TPAs).

Initial recruitment efforts involved email outreach to key companies, prioritising those with significant market share and regional influence. Where direct contact information was unavailable, further recruitment was facilitated through professional networking platforms, particularly LinkedIn, by identifying and reaching out to individuals in relevant roles (e.g., medical officers, policy managers, claims executives). In total, 26 companies were contacted:

- Five participants responded via email invitations.
- Four were recruited via LinkedIn contact and follow-up.
- The remaining three participants were identified through snowball sampling, where initial participants recommended colleagues from their professional networks.

Participant selection aimed to capture a diversity of medical and non-medical professional backgrounds, representing a range of institutional perspectives and levels of expertise in coverage policy, reimbursement evaluation, and health innovation assessment.

The final sample consisted of twelve participants, and interviews continued until data saturation was reached, i.e., when no new concepts or themes emerged from additional contextually grounded insights into the insurance sector’s perspectives on PGx adoption in the UAE [27]. No incentives were offered. Non-response was most commonly due to lack of organisational approval or time constraints, as reported in email/LinkedIn replies.

### Data collection

Data were collected between March and September 2024. The interviews followed a structured guide, allowing participants to express their views openly while enabling the interviewer to explore relevant themes based on expertise and role. Most interviews were conducted virtually via Microsoft Teams, while a few were held face-to-face at the participants’ offices.

Participants were initially contacted via personalised emails that included an information sheet, a consent form, and the interview guide. Reminder messages were sent one day and one hour before the scheduled interview to confirm attendance and facilitate preparation. The interview guide was shared at least 48 h in advance, allowing participants sufficient time to reflect on the discussion areas and prepare any points they wished to raise. The interview lasted 30–60 min, depending on the participant’s engagement and availability.

All interviews were conducted by a female PhD candidate in Public Health, with a background in clinical pharmacy and formal training in qualitative research. She had no prior relationship with any of the participants.

Reflexivity was embedded throughout the data collection process. The researcher maintained a reflexive journal, documenting methodological decisions, evolving interpretations, and any assumptions or concerns related to positionality during fieldwork. Open-ended questions and neutral prompts were used to encourage honest responses, whether supportive or sceptical of PGx adoption.

Credibility measures included member checking with two participants (to verify transcript accuracy), regular peer debriefings within the research team, and maintenance of an audit trail (versioned interview guide, codebook iterations, decision memos).

### Theoretical framework and data analysis

Data analysis proceeded in three interconnected layers. Firstly, the descriptive layer involved inductive thematic analysis to capture participants’ accounts in their own terms, allowing perspectives to emerge openly without predefined categories. This ensured that the full range of opinions—including subtle or unexpected insights—was not overlooked. A comparative analysis between executive- and middle-management participants was then conducted to highlight variations linked to organisational roles.

The second analytical layer applied two complementary theoretical frameworks to deepen the interpretation of the descriptive findings. Institutional Theory was used to examine external forces shaping insurer behaviour (mimetic, normative, and coercive pressures) [28]. At the same time, TAM illuminated internal decision-making factors, including perceived usefulness, ease of use, trust in the technology, and cost-utility [29]. Together, these frameworks provided a dual-lens approach that captured both macro-level structural forces and micro-level decision-making dynamics, enabling a more holistic understanding of the complexities of PGx implementation in a mixed public-private healthcare system like the UAE’s.

**Fig. 1:**
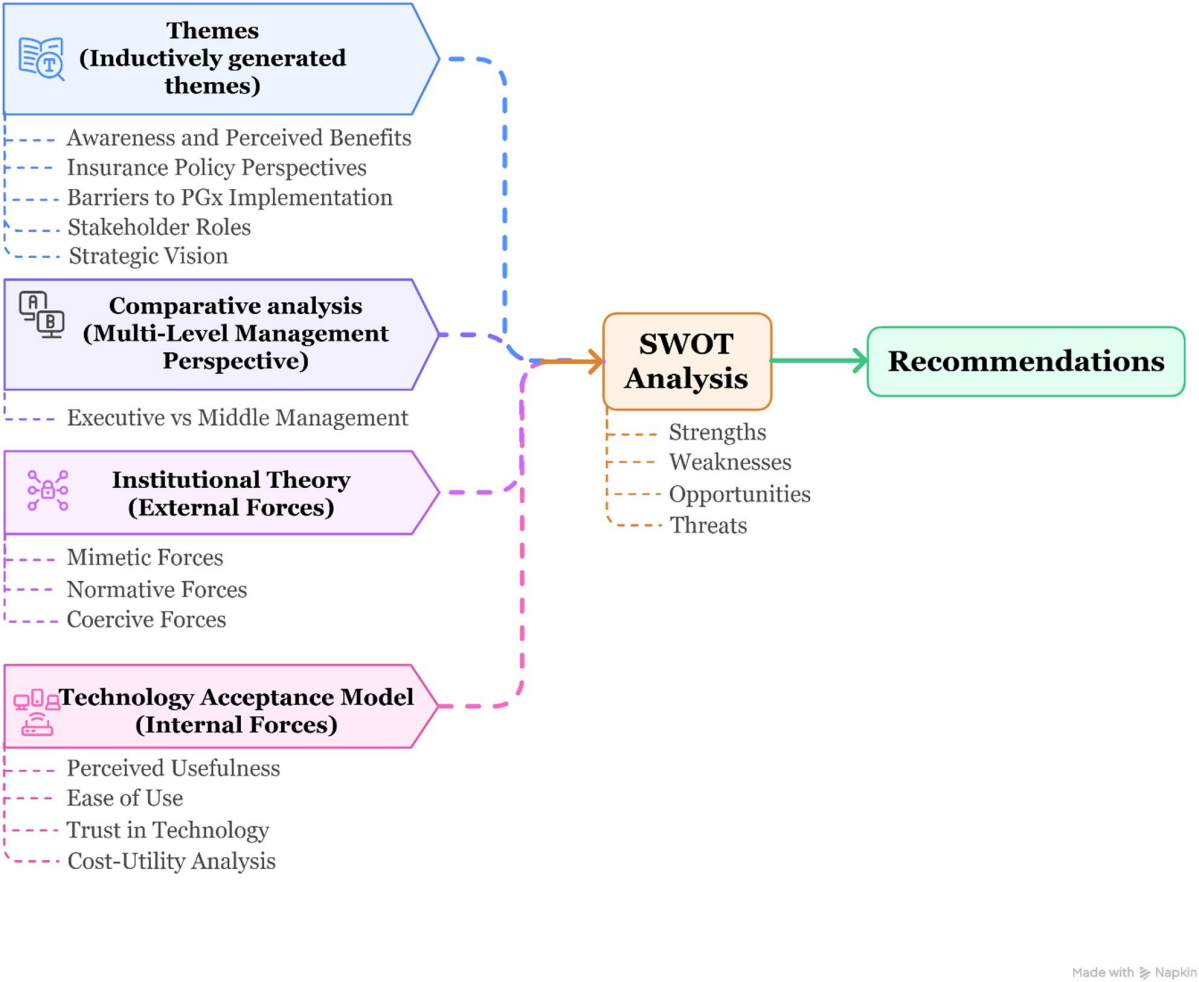
Summary of analytical method

The final analytical layer employed SWOT analysis to translate the thematic and theoretical insights into structured, actionable policy guidance. By organising findings into strengths, weaknesses, opportunities, and threats, the SWOT framework enabled a strategic synthesis that supports informed decision-making and planning for PGx integration within (Fig. 1) [30].

### Ethical consideration

The Social Sciences Research Ethics Committee of the UAEU granted ethical approval for this study under protocol number ERSC_2024_4212 on 1 March 2024. All participants received a detailed information sheet outlining the study’s objectives, procedures, voluntary nature, the right to withdraw at any stage without consequence, and confidentiality protections. Written informed consent was obtained prior to the start of each interview. To maintain confidentiality, all transcripts were anonymised, and participants were identified using only their initials.

## Results

### Participants’ demographics

Twelve in-depth interviews were conducted with insurance stakeholders representing both insurance companies and Third-Party Administrators (TPAs) across the UAE. Participants were evenly distributed between executive and middle management roles and included professionals from both medical (*n* = 7) and non-medical (*n* = 5) backgrounds. A majority (*n* = 8) had more than 15 years of experience in the insurance sector. While most participants were based in Dubai (*n* = 7), others were from Abu Dhabi and Al Ain. Notably, none of the stakeholders had received formal training in PGx, nor had any had direct experience with PGx testing in their professional context (Table 1).

**Table 1:** Participants’ demographics and professional characteristics

Characteristic		*N*
Age	≤ 45 years old	5
	> 45 years old	7
Gender	Female	3
	Male	9
Geographic Location	Dubai	7
	Abu-Dhabi	3
	Al-Ain	2
Educational Background	Medical	7
	Non-medical	5
Position Level	Executive Management	6
	Middle Management	6
Years of experience in the insurance sector	≤ 15 years	4
	> 15 years	8
Formal training in PGx	Yes	0
	No	12
Experience with PGx testing in the work context	Yes	0
	No	12

### Thematic analysis

**Fig. 2:**
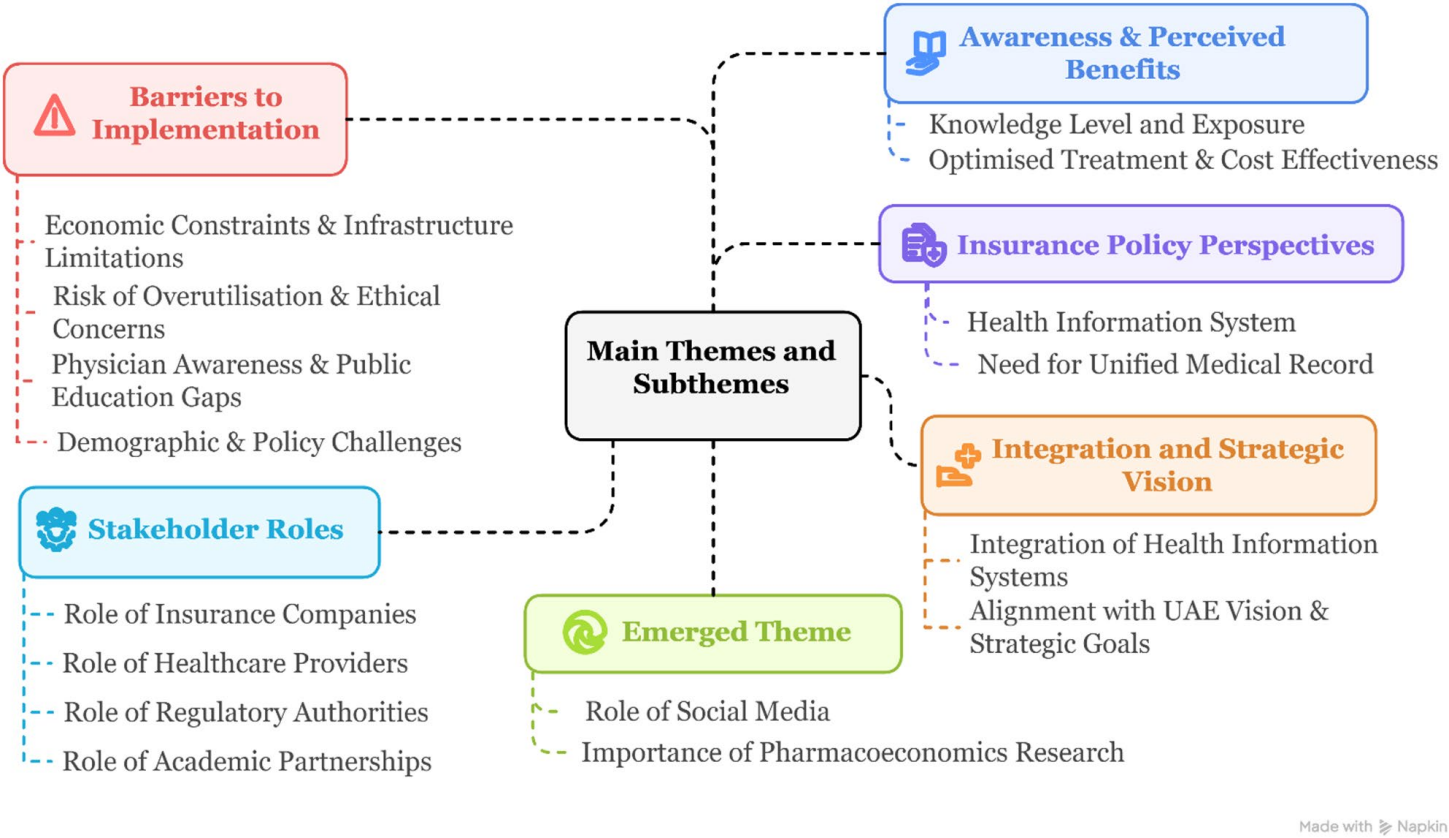
Thematic map of insurance stakeholders’ perspectives on PGx implementation

Thematic analysis of the interviews revealed five primary themes that reflect the complexity of PGx implementation from the perspective of insurance stakeholders in the UAE: (1) Awareness and Perceived Benefits (2), Insurance Policy Perspectives and Coverage Considerations (3), Barriers to Pharmacogenomics Implementation (4), Stakeholder Roles and Responsibilities and (5) Integration and Strategic Vision for Healthcare Systems (Fig. 2).

#### Theme 1

Awareness and Perceived Benefits of Pharmacogenomics.

This theme illustrates the evolving awareness of PGx among insurance stakeholders in the UAE and highlights its perceived potential to improve both patient outcomes and healthcare resource utilisation. While knowledge remains variable, participants demonstrated a growing recognition of PGx’s value, particularly in high-impact clinical contexts. This theme encompassed two subthemes.

### Knowledge level and exposure

Participants’ familiarity with PGx varied widely and was often influenced by their specific roles within the insurance sector. Those in clinical, underwriting, or medical claims positions showed greater awareness than those in purely administrative roles. However, even among those with awareness, most described only a basic understanding of PGx as a concept that combines pharmacology and genetics to guide drug selection.

*Quote “It helps understand whether the patient will have side effects in case they receive the medication” (M.F.).*

*Quote “My understanding… is that it is a combination of pharmacology and genomics… the efficacy is increased*,* and the side effects are decreased.” (A.W)*.

All participants highlighted the lack of formal training, workshops, or policy directives on PGx as key reasons for their limited knowledge of PGx. The field was often described as emerging, with minimal integration into routine insurance decision-making.

*Quote “No*,* pharmacogenomics application*,* not at all… Pharmacogenomics is a new field*,* especially here in the UAE. It is still under development” (M.F.).*

In several cases, participants’ understanding of PGx was either partial or conceptually conflated with broader genetic or oncological testing. For some, PGx was perceived mainly as a tool for predicting medication side effects, with little recognition of its broader clinical applications, such as dosing and therapy selection. Others associated it exclusively with specialised areas like cancer or biological therapies.

*Quote “I do not know all the details*,* but I have a general idea. It is related to oncology and biological treatments.” (H.H)*.

### Optimised treatment and cost effectiveness

Participants viewed PGx as a valuable tool for improving clinical decision-making, particularly in complex cases where conventional prescribing may be ineffective or risky, by enabling more precise drug and dose selection, reducing adverse reactions, and supporting better treatment adherence.

*Quote “If a medication has a high risk of side effects and the patient ends up not completing the treatment*,* it is a waste of time*,* money*,* and could even be life-threatening.” (H.H.)*

Beyond clinical value, participants recognised the economic potential of PGx in reducing avoidable costs. By enabling more efficient prescribing and minimising long-term medication use, PGx was seen as a way to improve health outcomes while limiting unnecessary healthcare expenditure.

*Quote “Better care outcomes for our insured members and savings from unnecessary utilisation of long-term medications that do not have the desired outcome.” (A.B.)*

Participants also discussed how PGx could influence coverage decisions by providing evidence of medical necessity, particularly in high-risk treatment plans. However, not all were convinced that insurers would directly benefit under current policy models.

*Quote “If a patient has prescribed X medication… and that test shows the side effect… then that can support the medical necessity coverage of the test.” (M.F.)*

*Quote “PGx testing may benefit patients by improving outcomes and reducing side effects*,* but insurance companies would not directly benefit.” (S.A.)*

#### Theme 2

Insurance Policy Perspectives and Coverage Considerations.

This theme examines how PGx is viewed through the lens of insurance coverage, highlighting concerns about feasibility, affordability, and policy alignment. Participants emphasised the need for clear criteria to guide PGx reimbursement and stressed the importance of prioritising high-cost, high-impact cases over routine applications. This theme encompassed two subthemes.

### Coverage criteria and plan disparities

Participants consistently called for well-defined coverage policies that justify PGx testing on the basis of clinical value and cost-effectiveness. They noted that while insurers are not inherently opposed to PGx, decisions must align with the member’s policy scope.

*Quote “We are not rejecting because of cost… It is about the coverage of the policy.” (K.O)*.

An apparent disparity was identified between high-premium and basic plans. Many felt PGx testing would be challenging to fund under low-cost policies due to the high upfront costs.

*Quote “For a basic plan… it would be tough for an insurer to cover such services.” (A.A)*.

*Quote “If somebody is spending more than the premium itself*,* then it will not be viable in a commercial sense for an insurance company.” (A.W)*.

Participants widely supported a selective coverage model that limits PGx to serious or high-cost medical conditions rather than extending it to chronic diseases with standard treatments.

*Quote “This test should be exclusive to serious medical conditions such as cancer and severe psychosis*,* not for common conditions like high blood pressure or cholesterol.” (R.O)*.

### Learning from international experiences

Some stakeholders suggested looking to global practices to inform PGx implementation in the UAE. International examples were seen as valuable references, provided they were adapted to local systems.

*Quote “The international guidelines could be used as a reference… putting the customisation or setting additional controls and rules… within the UAE needs to be created as well.” (P.A)*.

#### Theme 3

Barriers to Pharmacogenomics Implementation.

This theme outlines the practical, structural, and systemic obstacles to implementing PGx in the UAE. Participants identified affordability, infrastructure gaps, misuse risks, low awareness, and short-term health policy cycles as persistent barriers that must be addressed before PGx can be adopted at scale. This theme encompassed four subthemes.

### Economic constraints and infrastructure limitations

High testing costs and the UAE’s reliance on outsourced laboratory services were identified as key limitations to the adoption of PGx. Participants emphasised the need for alternative financing models and local infrastructure to mitigate long-term expenses.

*Quote “The main barrier is coverage only*,* from the payer’s side” (F.A)*.

Quote “The test cost is significantly higher when outsourced than in-house” (M.F.).

### Risk of overutilisation and ethical concerns

Several participants expressed concern over the potential overuse of PGx testing, driven by provider incentives or a lack of regulation. Ethical risks were also noted, including misuse of genetic data to influence insurance premiums, despite the UAE’s strong data protection laws.

*Quote “There is a real overutilisation here*,* driven by healthcare providers and the pharmaceutical industry.” (A.B.)*

Quote “There are chances of misuse… the premium plan gets modified accordingly.” (A.W.)

### Physician awareness and public education gaps

Limited awareness among physicians and patients was identified as a critical barrier. Participants emphasised the need to integrate PGx content into continuing medical education and to develop public-facing educational campaigns to enhance understanding and trust.

*Quote “I know doctors have a lot of continued medical education programs… I am unsure how many new strategies are integrated into the CME programs.” (A.W.)*

*Quote “Awareness has to be worked on*,* really*,* as a focus for the next few years.” (A.B.)*

### Demographic and policy challenges

The UAE’s transient population and the one-year insurance model were seen as incompatible with long-term investment in PGx. Participants noted that short-term contracts limit ROI and called for UAE-specific evidence to support phased implementation.

*Quote “The average stay in the UAE is not that high.” (M.S.O.)*

Quote “Short-term expat population impacts long-term cost-benefit analysis for insurer.” (A.A.)

*Quote “It is important to ensure studies are conducted in the UAE to gather evidence on the return on investment (ROI)*,* as the healthcare system here differs from other countries.” (S.A.)*

Insurers also highlighted the importance of long-term planning and expressed concern over the one-year policy cycle, which limits investment in preventive innovations, such as PGx.

Quote “Insurance contracts are one-year plans, so anything you need to embed… has to go hand in hand with one-year plans.” (M.S.O.)

#### Theme 4

Stakeholder Roles and Responsibilities.

This theme captures insurance stakeholders’ views on the roles of key actors in enabling PGx implementation in the UAE. Participants emphasised the need for coordinated efforts among insurers, healthcare providers, regulators, and academic institutions. From their perspective, successful PGx integration depends on shared accountability, policy alignment, and the generation of locally relevant evidence to support clinical and financial decision-making. This theme encompassed four subthemes.

### Role of insurance companies

From the insurers’ perspective, implementing PGx is pivotal yet complex. Participants emphasised that insurers must balance access to innovative technologies with the commercial realities of risk and sustainability. This includes adjusting policies, evaluating return on investment (ROI), and contributing to long-term system planning.

*Quote “Insurers have to make sure policies are adjusted to make access possible while still being financially viable.” (M.S.O.)*

Participants also viewed insurers as key actors in facilitating coverage decisions, particularly through well-designed reimbursement models that align with both clinical benefit and financial risk mitigation.

*Quote “Reimbursement policies must reflect the actual value of these tests for patients and the healthcare system.” (M.F.)*

Some participants expressed that insurers are often expected to lead without sufficient guidance, highlighting the need for clear frameworks and shared responsibility. They underscored that insurers alone cannot drive adoption without regulatory backing and clinical leadership.

*Quote “Bringing all stakeholders together to work at one table will be a bigger challenge.” (M.S.O.)*

### Role of healthcare providers

Insurers consistently identified physicians as the central gatekeepers for PGx adoption. Their clinical endorsement was viewed as essential for building patient trust, justifying the test, and ensuring ethical use. Participants highlighted that without physician leadership, PGx is unlikely to gain traction among patients or be accepted as part of routine care.

*Quote “Physicians should be part of the task force… advising on how to do this comprehensively.” (P.A.)*

Participants also suggested that physicians have a dual responsibility: to educate patients about the test’s relevance and to guide insurance approvals by framing PGx within evidence-based treatment plans. Their role was seen as both clinical and strategic.

*Quote “The role to implement and create demand should be from the physician’s side. If the physician suggests or recommends this as a treatment plan*,* the patient and the insurance company will follow.” (A.B.)*

### Role of regulatory authorities

Insurance stakeholders repeatedly emphasised that regulatory authorities, particularly the Dubai Health Authority (DHA) and Department of Health (DOH), are the primary enablers of PGx implementation in the UAE. Participants viewed regulatory mandates as a prerequisite for action, noting that insurers are unlikely to support widespread coverage without top-down policy alignment.

*Quote “The health authority mandates will play a significant role in whether insurance companies agree to approve the pharmacogenomic test.” (R.O.)*

*Quote “The health authorities are the main and key players in this equation because if they mandate it*,* everyone will follow.” (A.P.)*

Participants emphasised the need for regulators to issue specific, enforceable guidelines that clarify when and for whom PGx testing should be utilised. This regulatory clarity was deemed essential to prevent misuse, ensure consistent coverage decisions, and establish trust in the system.

*Quote “We require very specific guidelines on which conditions and which situations this would be utilised for.” (A.B.)*

In addition to mandate-setting, regulators were also seen as responsible for embedding PGx into broader healthcare reforms. The DHA’s Ejadah initiative (a value-based healthcare programme designed to align care delivery with measurable outcomes) was viewed as a promising example of value-based care alignment that could support PGx integration.

*Quote “The health authorities have started moving toward value-based healthcare… This initiative could support the effective implementation of pharmacogenomics by aligning healthcare practices with patient-centred*,* outcome-focused care.” (M.S.O.)*

### Role of academic partnerships

Insurers strongly emphasised the role of academic institutions in providing the local evidence needed to support PGx implementation. Participants noted that most existing studies are based on healthcare models that do not accurately reflect the UAE context, thereby limiting their applicability to insurance decision-making.

*Quote “The healthcare system model in the UAE differs from other areas and regions… so their cost-effectiveness studies are not applicable.” (F.A.)*

Quote “We need research specifically discussing the costs and benefits of implementing pharmacogenomics in the UAE healthcare system.” (R.O.)

Participants envisioned academic institutions not only as research generators but also as strategic advisors, helping tailor PGx guidelines to the country’s evolving healthcare priorities.

*Quote “We need partnerships between academic institutions and insurance companies for better decision-making.” (A.W.)*

#### Theme 5

Integration and Strategic Vision for Healthcare Systems.

This theme presents the views of insurance stakeholders on the future direction of PGx within the UAE healthcare system. Participants emphasised the importance of integrating PGx into the digital health infrastructure and aligning its implementation with national strategies for innovation, efficiency, and patient-centred care. This theme encompassed two subthemes.

### Integration of health information systems

Participants strongly supported linking PGx results with national electronic health record systems, such as NABIDH and MALAFFI. They viewed this integration as essential to reducing test duplication, enabling clinical decision-making, and detecting overutilisation. Centralised access to patient history was also seen as a safeguard against fraud.

*Quote “NABIDH connects all electronic medical records of all healthcare providers.” (M.F.)*

*Quote “Imagine if we had that! It would significantly reduce abuse and fraud*,* as a complete medical history would be readily accessible.” (H.H*.)

### Alignment with UAE vision and strategic goals

Insurers recognised that PGx aligns with the UAE’s broader national agenda for innovation, precision medicine, and sustainable healthcare. PGx was seen as a natural extension of the country’s commitment to high-impact technologies such as AI and genomics.

*Quote “The future is bright*,* with breakthroughs coming*,* especially with A.I.’s use.” (A.W.)*

*Quote “The UAE’s vision for innovation and sustainability aligns with implementing new technologies like pharmacogenomics*,* which can help improve patient outcomes.” (S.A.)*

*Emerged themes*

In addition to the core thematic categories, two cross-cutting insights emerged from participants’ reflections. These themes highlight the broader contextual factors that may influence the successful implementation of PGx.

*The role of social media in patient awareness*

Participants recognised the dual influence of social media as both a powerful educational tool and a potential source of misinformation. While its accessibility was seen as a facilitator for raising awareness and promoting patient acceptance of PGx, participants cautioned against unregulated content and emphasised the need for strategic communication efforts.

*Quote “Social media is a great platform to educate anyone*,* so it can definitely help in educating patients.” (A.A.)*

*Importance of pharmacoeconomics research*

The need for locally relevant pharmacoeconomic evidence was repeatedly emphasised. Participants viewed this as essential for informing policy decisions, supporting insurer confidence, and guiding cost-effective implementation. They called for research models that account for the UAE’s unique demographics and healthcare financing structures.

*Quote “We have to start with the pharmacoeconomic research and modelling.” (A.B.)*

*Quote “We need cost studies*,* cost-minimising studies*,* and cost-effectiveness studies*,* specifically within the UAE context.” (S.A.)*

#### Comparative matrix analysis

This analysis was conducted to explore whether meaningful differences or convergences existed between senior (high-level) and operational (middle-level) insurance decision-makers in their perspectives on PGx implementation. Recognising the layered structure of healthcare decision-making, this comparative matrix was designed not only to illustrate how strategic versus operational roles shape priorities and perceived challenges, but also to underline the importance of involving stakeholders across organisational hierarchies in shaping implementation strategies.

High-level stakeholders, typically senior executives or department heads, are focused on system-wide priorities such as the need for national guidelines, evidence of cost-effectiveness specific to the UAE context, and phased implementation targeting high-impact therapeutic areas like oncology. Their insights reflected long-term considerations, including alignment with the UAE’s healthcare innovation agenda, the potential for reducing hospitalisation costs, and the integration of PGx into broader value-based care frameworks.

In contrast, middle-level stakeholders, who are more directly involved in operationalising insurance policies, tended to prioritise day-to-day implementation concerns. These included logistical challenges such as testing availability, the need for laboratory infrastructure, physician awareness, and the integration of PGx results into existing electronic workflows. Their focus was on the practical implications of PGx at the provider–patient level and how they would fit within current insurance and claims systems.

Despite these differing emphases, both groups shared concerns about the risks of overutilization, test affordability, and the need for regulatory alignment. Ethical and legal concerns were not perceived as significant barriers, primarily because of trust in existing data protection frameworks; however, some high-level respondents called for PGx-specific safeguards (Table 2).

Overall, the analysis demonstrates that bridging strategic vision with operational feasibility is crucial to the successful implementation of PGx. Engaging both high- and middle-management stakeholders ensures that implementation plans are not only visionary but also grounded in the realities of clinical and insurance system workflows.

**Table 2:** Comparison of higher and middle management perspectives on key aspects of PGx testing policy implementation

Aspect	Higher management	Middle management	Common concerns	Key differences
Barriers to Implementation	✓ Concerns regarding short-term insurance cycles and transient populations ✓ Lack of UAE-specific cost-effectiveness data ✓ Risk of overutilization without structured policies	✓ High test costs and limited lab infrastructure ✓ Insufficient provider awareness and public acceptance ✓ Lack of in-house lab capacity	Overutilization and high cost are central barriers	Higher management emphasised systemic policy and evidence gaps, while middle management focused on operational limitations and clinical readiness
Policy and Coverage Considerations	✓ Policies exclude PGx testing unless mandated by regulators ✓ Advocated for UAE-specific clinical guidelines	✓ Coverage often limited to approved treatment contexts ✓ Preventive applications are rarely reimbursed	Agreement on the need for alignment with clinical guidelines and regulatory input	Higher management emphasised top-down policy directives, while middle management preferred flexible, case-based approvals.
ROI and Cost-Effectiveness	✓ Called for local pharmacoeconomic studies to demonstrate ROI ✓ Highlighted long-term benefits from adverse event reduction	✓ Stressed prevention benefits in oncology settings ✓ Concerned about evaluating ROI within annual policy terms	Both stakeholder levels highlighted the need for cost-benefit evidence	Higher management took a long-term, system-wide perspective, while middle management focused on immediate clinical returns
Stakeholder Roles	✓ Health authorities should lead PGx policy development ✓ Academia must generate local implementation evidence ✓ Physician leadership is pivotal to clinical uptake	✓ Physicians identified as key decision influencers ✓ Researchers should support evidence-based policy decisions ✓ Insurers rely on regulatory clarity	Consensus on the role of regulators, physicians, and researchers in advancing PGx	Higher management focused on structured governance, while middle management emphasised field-level advocacy and data generation.
Ethical and Legal Concerns	✓ Suggested additional safeguards for PGx-specific data ✓ Emphasised the need for transparency in genomic data handling	✓ Expressed confidence in existing UAE data protection systems ✓ Privacy standards seen as adequate	Shared understanding of confidentiality as a critical issue	Higher management proposed additional ethical oversight, while middle management viewed current protections as sufficient
Potential for Overutilization	✓ Expressed concern over unregulated test requests ✓ Called for the enforcement of clinical indication criteria	✓ Echoed the need for restrictions to avoid unnecessary testing ✓ Flagged financial incentives as potential misuse drivers	Both levels advocated for clear guidelines to mitigate misuse	No significant divergence; strong alignment in overutilization concerns

## Discussion

### General insights on PGx adoption in the UAE from the insurer’s perspective

This study integrates insurers’ perspectives on PGx adoption in the UAE. Participants consistently acknowledged PGx’s potential to personalise treatment, reduce adverse drug reactions, and lower long-term healthcare costs. However, this optimism was tempered by a range of systemic and operational barriers.

Core constraints included high testing costs, uncertainty around return on investment (ROI), and concerns about overutilisation. Some of these challenges, such as economic feasibility concerns and integration difficulties, are widely reported across international health systems. Others were distinctly UAE-specific, including the divide between premium and basic insurance plans, inequities in access, and the transient nature of the population with short-term residency and annual policy renewals, which limits the feasibility of long-term cost-effectiveness evaluations.

These patterns reflect a dual reality: while the UAE shares many global barriers to PGx adoption, it also faces unique contextual constraints shaped by its insurance structures, demographic profile, and market dynamics [21, 31–33].

### Diagnostic interpretation using Institutional Theory and TAM

This section interprets the findings through two complementary theoretical lenses, Institutional Theory and the Technology Acceptance Model (TAM), to explain why insurers in the UAE respond to PGx as they do. While the thematic analysis outlined insurers’ concerns, expectations, and perceived barriers, these frameworks help illuminate the broader system-level pressures and internal evaluation processes that shape those views. The dual-lens approach clarifies why insurers appear cautious despite acknowledging PGx’s potential.

#### Technology acceptance model (TAM)

**Fig. 3:**
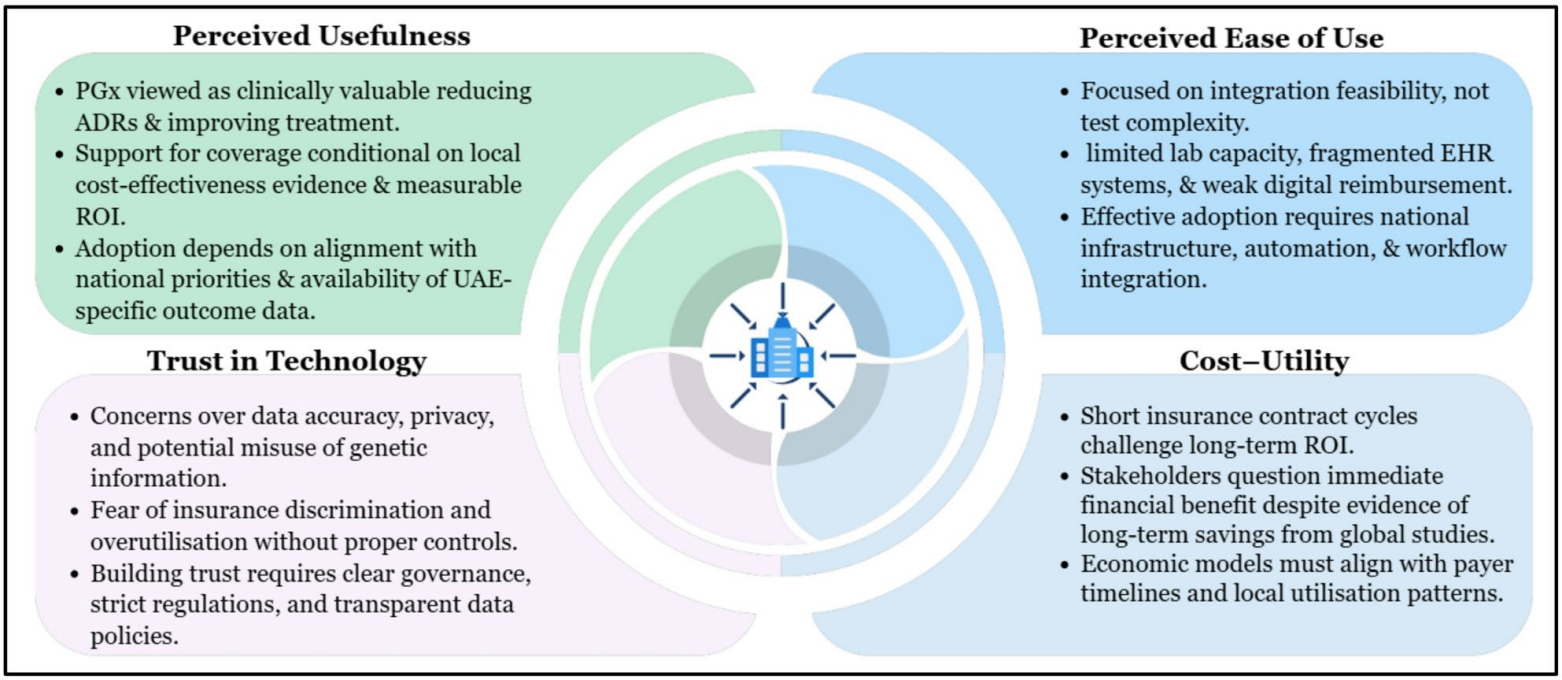
Technology acceptance model constructs shaping insurers’ evaluation of PGx adoption

The TAM explains technology adoption through two key constructs, Perceived Usefulness (PU) and Perceived Ease of Use (PEOU), which predict intention and system use. For complex health technologies such as PGx, TAM is often extended with additional constructs, including Trust in Technology and Cost-Utility, to address concerns about data integrity, clinical validity, financial sustainability, and contract constraints [29, 39, 40]. This extended framework was applied to interpret UAE insurers’ views, offering a more nuanced understanding of internal readiness and decision-making (Fig. 3).

*Perceived usefulness (PU)*

Perceived Usefulness reflects stakeholders’ beliefs about whether a technology delivers meaningful clinical and system-level benefits. Insurers in the UAE acknowledged the clinical value of PGx, consistent with international evidence showing reduced adverse drug reactions, improved therapeutic precision, and support for evidence-based prescribing [41]. However, their assessment of usefulness extended beyond clinical outcomes to whether PGx could offer tangible financial value. Support for coverage was therefore contingent on locally generated cost-effectiveness evidence and on clarity about how PGx aligns with national health priorities and insurers’ long-term return on investment. In essence, insurers recognised PGx’s potential but remained cautious without UAE-specific evidence demonstrating that both its clinical benefits and financial implications justify adoption [42].

*Perceived ease of use (PEOU)*

Perceived Ease of Use reflects how readily stakeholders believe PGx can be integrated into existing systems, with operational complexity often hindering adoption, regardless of clinical usefulness [43, 44]. In this study, insurers’ concerns centred not on the test itself but on the surrounding infrastructure: ecosystem readiness, electronic health record linkage, laboratory capacity, workflow efficiency, and reimbursement processes. International evidence reinforces these issues; poor integration with clinical decision support has impeded uptake in Europe, whereas structured workflows and strong clinical leadership have facilitated it [10, 45]. In the UAE, limitations in digital interoperability and system integration suggest that operational readiness—and thus insurer confidence in routine PGx use will remain constrained without substantial infrastructure development [18].

*Trust in technology*

Trust emerged as a central determinant of insurers’ willingness to support PGx, which aligns with TAM’s emphasis on confidence in the underlying technology as a prerequisite for adoption. Concerns about test accuracy, the potential for inappropriate prescribing, and the handling of sensitive genetic information reflect deeper uncertainties about the robustness of governance structures surrounding PGx. These issues mirror international findings, where fears of discrimination, inadequate consent processes, and unclear data-protection mechanisms have consistently hindered genomic implementation [46, 47]. In the UAE context, stronger PGx-specific safeguards and clearer regulatory oversight are needed to build the trust required for safe and equitable implementation.

*Cost‑utility analysis*

Cost-utility was a major concern, as insurers questioned whether the high upfront costs of PGx could be justified within short insurance contract cycles and a highly mobile insured population. This reflects a broader global pattern in which payers must absorb immediate costs while clinical and economic benefits accrue gradually, even though a systematic review of 108 economic evaluations across 39 gene–drug pairs found that 71% of PGx interventions were cost-effective or cost-saving [48, 49]. Decision-analytic models from Europe and Asia further indicate that PGx-guided prescribing can reduce hospitalisations and prevent serious drug-related harm [50]. However, results across settings remain heterogeneous, reinforcing insurers’ view that robust, UAE-specific economic models are needed before PGx can be routinely incorporated into reimbursement structures.

Viewed through TAM, insurers’ cautious stance reflects a broader evaluation of feasibility, trust, and financial value, rather than a lack of recognition of PGx’s clinical potential.

#### Institutional theory

**Fig. 4:**
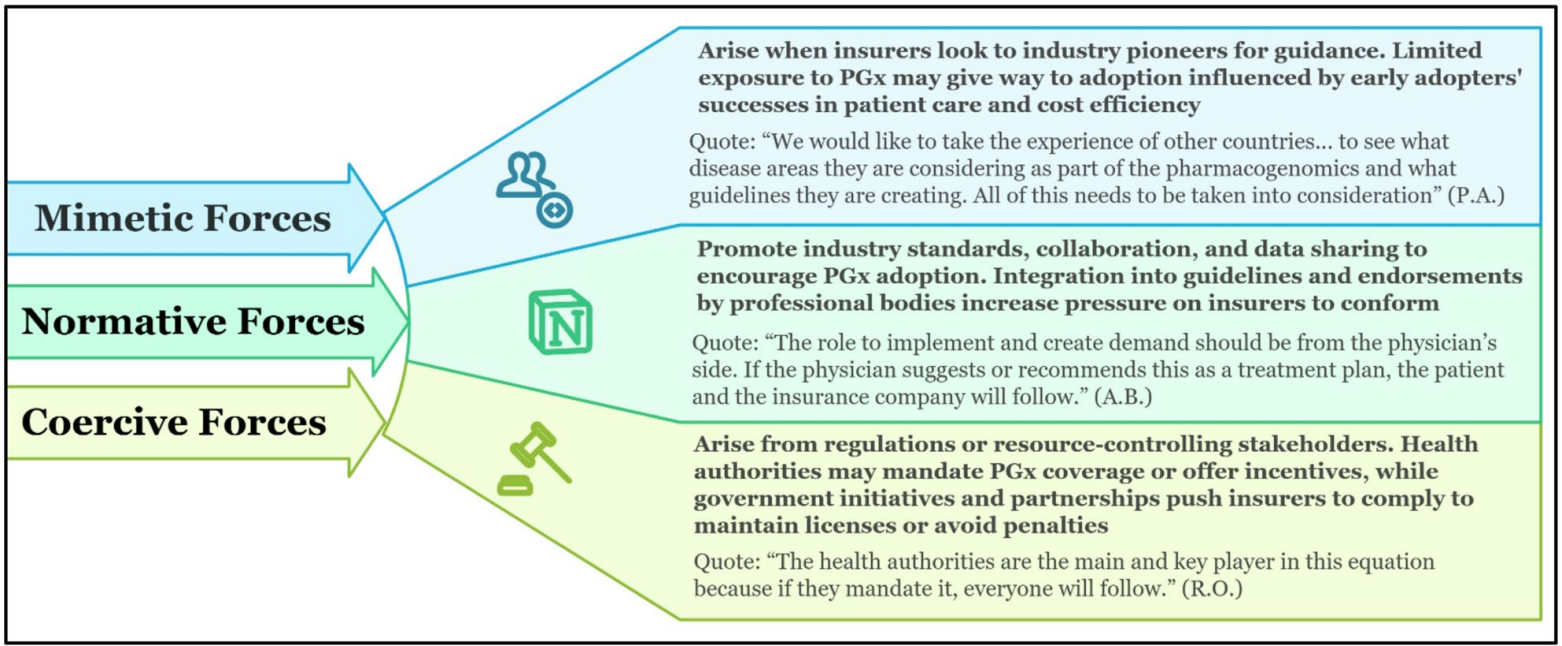
Institutional theory: external forces influencing PGx adoption among UAE insurers

Institutional theory suggests that organisational behaviour is influenced not only by internal goals or efficiency but also by the broader institutional environment, which exerts coercive, normative, and mimetic pressures [28, 34]. These external forces help explain how UAE insurers respond to evolving expectations around PGx adoption and influence their perceived readiness to integrate such innovation (Fig. 4).

*Corecive forces*

Coercive pressures arise from formal policies, legal mandates, and governmental expectations. Viewed through this lens, insurers’ reluctance to support PGx reflects weak top-down direction in the UAE. Participants’ calls for clear national guidelines indicate that, without mandated frameworks, insurers perceive little institutional obligation to integrate PGx. This pattern aligns with international experiences: in England, early PGx implementation stalled due to unclear roles and fragmented responsibility, whereas at the Mayo Clinic, adoption accelerated only after formal guidance and required certification were introduced. Chen et al. (2023) similarly found that inpatient engagement remained limited until policy and system integration were established [35–37].

*Normative forces*

Normative pressures arise from shared professional expectations and clinical standards. In the UAE, insurers’ reliance on physician leadership, endorsement from national medical societies, and the existence of clear clinical guidelines reflect their need for normative validation before committing to PGx coverage. International experience similarly shows that clinician confidence, pharmacist training, interdisciplinary collaboration, and the presence of PGx “champions” are central to building normative legitimacy for implementation [36–38]. Structured models, such as Mayo Clinic’s PGx framework, demonstrate how internal professional endorsement can sustain adoption even as regulatory mandates evolve. For the UAE, this suggests that strengthening clinician engagement and embedding PGx within established care pathways may be just as important as regulatory reform.

*Mimetic forces*

Mimetic pressures occur when organisations imitate practices from perceived leaders to reduce uncertainty. Insurers in this study expressed interest in international PGx models and in early-adopter countries, viewing them as potential templates for implementation in the UAE. However, this interest remains largely aspirational, as local contextual differences financing structures, regulatory processes, and demographic profiles limit the direct transferability of foreign models. International literature shows that mimicry supports adoption only when organisations can adapt external practices to their own institutional conditions [38]. In the UAE, the absence of such adaptation mechanisms explains why international models influence insurers conceptually but have not yet translated into operational change.

Institutional Theory, therefore, helps explain why insurers’ decisions align closely with the broader regulatory, professional, and structural contexts in which they operate.

### Strategic analysis for PGx adoption in the UAE using SWOT analysis

**Fig. 5:**
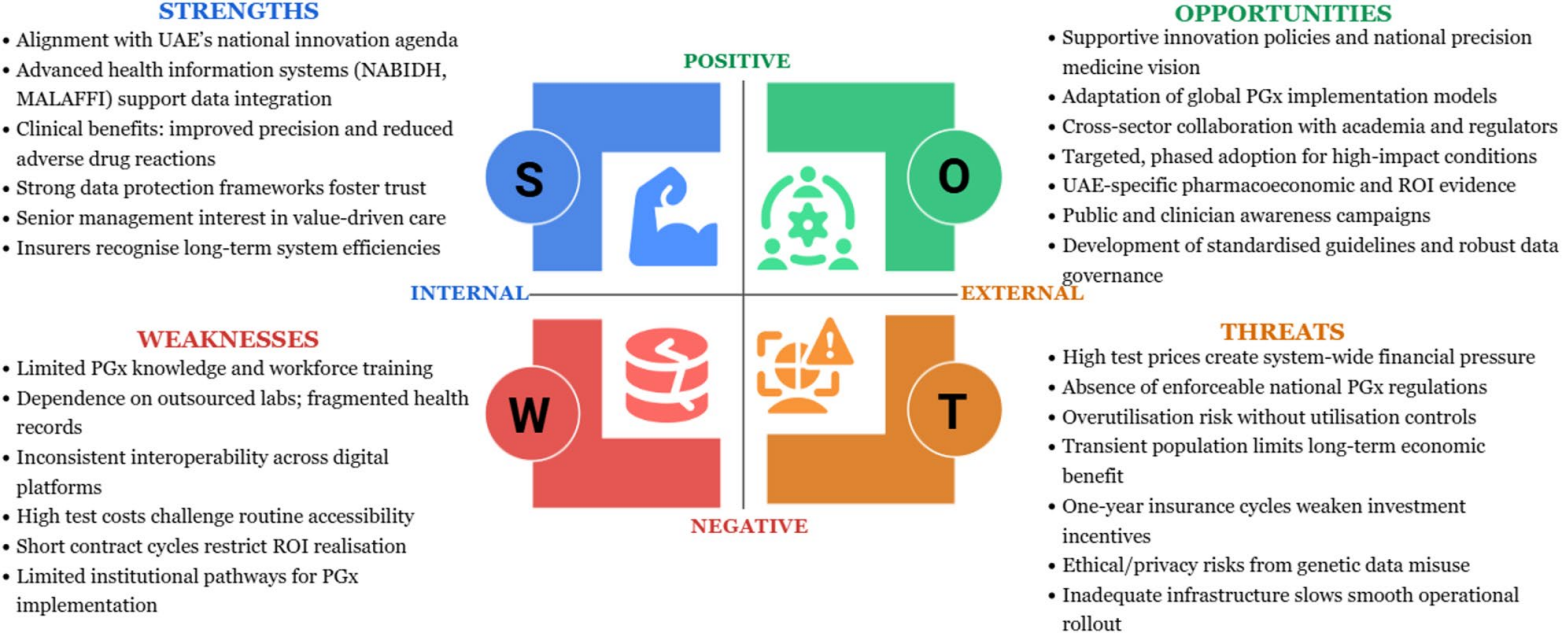
SWOT analysis for PGx adoption in the UAE Healthcare System

This SWOT analysis integrates the study’s findings with international evidence to highlight system-level conditions that can support or hinder PGx implementation in the UAE (Fig. 5) [30].

*Strengths*

Insurers recognised the clinical value of PGx in improving treatment precision, reducing ADRs, and potentially lowering long-term healthcare costs—benefits well supported by international evidence showing fewer treatment failures and preventable hospitalisations [21, 33, 51]. The UAE’s investment in digital health platforms such as NABIDH and MALAFFI offers a technical foundation for integrating PGx into electronic health systems, reflecting global findings that EHR-embedded PGx data improve uptake and clinical efficiency [41, 52, 53]. Moreover, the UAE’s strong data protection legislation fosters trust in genomic data governance, and its growing emphasis on precision medicine positions PGx as a natural fit for emerging national health priorities [54].

*Weaknesses*

Despite these enablers, insurers face substantial structural barriers that weaken the business case for PGx. High testing costs, uncertainty around long-term ROI, and high member turnover mirror global payer concerns [21, 33]. This financial mismatch, where upfront costs are borne immediately but benefits may not emerge for years, was described as a key deterrent to investment.

The mixed public–private insurance environment complicates unified decision-making and slows policy harmonisation [55]. Regulatory and clinical governance gaps were also repeatedly raised. Stakeholders emphasised the urgent need for clear UAE-specific national guidelines, standardised testing protocols, and ethical safeguards to ensure consistent and equitable adoption [56]. Workforce readiness remains another constraint, with clinicians lacking adequate PGx training and institutions having limited operational capacity [33, 57, 58]. Finally, incomplete system interoperability and remarkably inconsistent adoption of NABIDH and MALAFFI restrict the efficient integration of PGx into clinical workflows [41].

*Opportunities*

Stakeholders expressed a willingness to collaborate, learn from international best practices, and pursue phased adoption strategies. This includes adapting successful PGx frameworks from countries with mature programs, which could help reduce uncertainty and accelerate uptake. Such an approach would allow the UAE to mitigate financial and operational risks while building local capacity.

A critical opportunity lies in generating UAE-specific pharmacoeconomic evidence to guide policy and coverage decisions. The absence of such data was repeatedly cited as a barrier, particularly in the context of the transient UAE population and short insurance cycles. Regional evidence shows that GCC economic evaluations often lack methodological rigour, omitting societal perspectives, uncertainty modelling, discounting of future benefits, and real-world outcome capture [57, 58]. By addressing these gaps through robust local research, insurers could access concrete ROI data that strengthens their business case [18, 59]. The UAE’s culturally diverse and digitally connected population provides fertile ground for innovative awareness and education initiatives. Interactive, digitally enabled campaigns can facilitate two-way engagement between insurers, providers, and patients, enhancing acceptance of PGx [54].

Finally, the UAE’s status as a regional healthcare innovation hub offers a strategic opportunity to establish itself as a leader in genomic medicine implementation in the Middle East, attracting investment, partnerships, and global recognition. However, stakeholders also cautioned that without careful management, several external pressures could undermine these opportunities and erode confidence in PGx adoption.

*Threats*

The UAE’s transient population and annual insurance policy cycles were repeatedly identified as threats to PGx’s financial sustainability. This dynamic creates a misalignment between investment and returns, where insurers may bear the costs without retaining members long enough to benefit from reduced healthcare utilisation [60].

Overutilisation risk was another primary concern. Without clear utilisation frameworks, financial incentives in the UAE insurance market could drive unnecessary testing, inflating costs and undermining payer trust. Evidence from fee-for-service models shows that overuse can divert resources, create downstream interventions, and erode the value of even clinically effective technologies [61–63]. Ethical and data governance gaps, particularly the lack of enforceable national guidelines, pose additional threats. While insurers generally trust UAE data protection frameworks, they warn that any misuse of genetic data, such as discriminatory pricing or exclusions, could quickly erode trust [21].

Finally, fragmented coordination among UAE stakeholders with insurers, providers, regulators, and academic institutions working in isolation remains a systemic risk. Without multi-sectoral governance mechanisms, PGx adoption risks being uneven, with isolated pilot programmes failing to scale.

### Study strengths and limitations

This study offers timely and much-needed insights into insurance stakeholders’ perspectives on PGx implementation in the UAE—an angle largely absent from existing research. A key strength is the use of semi-structured interviews, which enabled thoughtful exploration of both strategic and operational considerations. Including participants from different organisational levels and professional backgrounds added depth, allowing us to compare perspectives shaped by varied roles and responsibilities. Analytical rigour was strengthened through a layered approach that moved from inductive thematic and comparative analysis to interpretation using Institutional Theory and TAM, and finally to a strategic synthesis through SWOT. Together, these methods served as a form of triangulation, enhancing the credibility and richness of the findings.

At the same time, some limitations should be acknowledged. As a qualitative study grounded in the UAE insurance context, the findings reflect the perspectives of a specific stakeholder group. They may not capture the full diversity of views across the sector. Participation was voluntary, and while the sample included both executive and middle-management representatives, additional organisational tiers may have offered further nuance. Interview data are also self-reported and subject to individual recollection and professional framing. Future research could include a broader range of stakeholders or apply additional analytical lenses to extend the transferability of these insights, particularly to countries with similar mixed public–private insurance systems.

## Conclusions and recommendations

This study shows that while insurers recognise the clinical promise of PGx, their willingness to support implementation is shaped by financial uncertainty, operational constraints, and the lack of clear regulatory direction. Strengthening PGx integration will depend on establishing UAE-specific evidence on cost-effectiveness, developing clear government-led coverage guidelines, and investing in educational initiatives that build clinicians’ and the public’s confidence and competency. Collaborative engagement among insurers, providers, regulators, and academic institutions will be critical to aligning incentives and creating an implementation environment that supports responsible, sustainable PGx adoption.

The implications extend beyond the UAE. Health systems with mixed public–private insurance structures, mobile populations, and short insurance cycles face similar challenges, making these findings relevant to policymakers across the Gulf region and other middle-income settings. Future research should expand to include a broader range of stakeholders and larger, more diverse samples to inform phased, scalable approaches tailored to regional healthcare financing and demographic realities.

**Operational definitions**

- Cost-Utility: Assesses the cost of an intervention relative to its health outcomes, typically measured in quality-adjusted life years (QALYs) or disability-adjusted life years (DALYs).
- Cost-Effectiveness: Compares the costs and outcomes of different interventions to identify the most efficient use of resources.
- Return on Investment (ROI): A financial metric that evaluates the profitability of an investment by comparing net benefits to initial costs.
- NABIDH: is a unified medical record developed by the Dubai Health Authority (DHA), connecting Dubai healthcare facilities and providing access to real-time patient records.
- MALAFFI: is a unified medical record developed by Abu Dhabi’s Health Information Exchange (HIE), which connects Abu Dhabi healthcare facilities and provides access to real-time patient records.
- DHA Ejadah: A value-based healthcare initiative launched by the Dubai Health Authority to improve care quality and align reimbursement with patient-centred outcomes.

## Supplementary Information

Supplementary Material 1

## Data Availability

The data supporting the findings of this study are available from the corresponding author upon reasonable request.

## Abbreviations

UAE: United Arab Emirates
PGx: Pharmacogenomics
NABIDH: National Unified Medical Record
CPIC: Clinical Pharmacogenetics Implementation Consortium
DPWG: Dutch Pharmacogenetics Working Group
CPNDS: Canadian Pharmacogenomics Network for Drug Safety
RNPGx: French National Network of Pharmacogenetics
ROI: Return on Investment
DHA: Dubai Health Authority
DOH: Department of Health
AI: Artificial Intelligence
COREQ: Consolidated Criteria for Reporting Qualitative Research
TPA: Third-Party Administrator
EHR: Electronic Health Records
TAM: Technology Acceptance Model
PU: Perceived Usefulness
PEOU: Perceived Ease of Use
SWOT: Strengths, Weaknesses, Opportunities, Threats
