# Supplementary material for: Strategic insights into pharmacogenomics coverage: a theory-informed SWOT analysis of UAE insurance stakeholders’ perspectives
Source: Hum Genomics. 2025 Dec 29;20:26. doi: 10.1186/s40246-025-00896-6 (PMC12860024; doi:10.1186/s40246-025-00896-6)
Supplement: Supplementary file 1 — Supplementary Material 1 [file 40246_2025_896_MOESM1_ESM.docx]

**Supplementary Materials**

***Table S1: COREQ Checklist***

| Domain | Topic | Item No. | Description | Reported |
| --- | --- | --- | --- | --- |
| Domain 1: Research Team and Reflexivity | Personal Characteristics | 1 | Which author/s conducted the interview or focus group? | Maram O Abbas |
|  |  | 2 | What were the researcher’s credentials? | MSc |
|  |  | 3 | What was their occupation at the time of the study? | Student |
|  |  | 4 | Was the researcher male or female? | Female |
|  |  | 5 | What experience or training did the researcher have? | Comprehensive training in qualitative methods, including thematic analysis and interview experience. |
|  | Relationship with Participants | 6 | Was a relationship established prior to study commencement? | No |
|  |  | 7 | What did the participants know about the researcher? | Academic background, study objectives, impact on healthcare and insurance policies. |
|  |  | 8 | What characteristics were reported about the interviewer/facilitator? | Background in public health, qualitative research experience. |
| Domain 2: Study Design | Theoretical Framework | 9 | Methodological orientation stated to underpin the study? | Thematic analysis, Institutional Theory, TAM, and SWOT analysis. |
|  | Participant Selection | 10 | How were participants selected? | Purposive, convenience |
|  |  | 11 | How were participants approached? | Online meetings |
|  |  | 12 | How many participants were in the study? | 12 |
|  |  | 13 | How many people refused to participate or dropped out? Reasons? | 5 (scheduling conflicts) |
|  | Setting | 14 | Where was the data collected? | Virtually via Microsoft Teams |
|  |  | 15 | Was anyone else present besides the participants and researchers? | No |
|  |  | 16 | Important characteristics of the sample? | Aged 30–55, roles in executive and middle management, medical/non-medical backgrounds, UAE insurance sector. |
|  | Data Collection | 17 | Were questions/prompts provided? | Yes, developed from literature and expert feedback. |
|  |  | 18 | Were repeat interviews carried out? | No |
|  |  | 19 | Was audio/visual recording used? | Yes |
|  |  | 20 | Were field notes made? | No |
|  |  | 21 | Duration of interviews/focus groups? | 30-40 minutes |
|  |  | 22 | Was data saturation discussed? | Yes |
|  |  | 23 | Were transcripts returned to participants for comment and/or correction? | Yes (2 participants) |
| Domain 3: Analysis and Findings | Data Analysis | 24 | Number of data coders? | 2 |
|  |  | 25 | Was a coding tree description provided? | Yes |
|  |  | 26 | Were themes identified in advance or derived from the data? | Derived from data |
|  |  | 27 | What software was used to manage the data? | NVivo |
|  |  | 28 | Did participants provide feedback on the findings? | No |
|  | Reporting | 29 | Were participant quotations presented to illustrate findings? | Yes |
|  |  | 30 | Was there consistency between data and findings? | Yes |
|  |  | 31 | Were major themes clearly presented? | Yes |
|  |  | 32 | Were minor themes described or discussed? | Yes |

***Table S2: Interview Guide***

| Section 1: Demographics | | | |
| --- | --- | --- | --- |
| 1. Gender | | | |
| 1. Age | | | |
| 1. Highest Education Level | | | |
| 1. Field of education | | | |
| 1. Name of the insurance company | | | |
| 1. Insurance coverage within the Emirates | | | |
| 1. Total years of experience in the insurance field | | | |
| 1. Total experience in the current company | | | |
| 1. Previous position | | | |
| 1. Current position | | | |
| 1. Type of the insurance company | | | |
| 1. Have you ever attended or been involved in workshops related to pharmacogenomics? | | | |
| 1. Have you ever suffered of medication adverse reaction or any of your family? | | | |
| 1. Have you participated in task forces or been involved in decision-making processes related to pharmacogenomic testing? | 1. Which committee were you collaborating with? 2. What type of task force? 3. What was your role? 4. What was the discussed proposal? 5. What was the outcomes if there is at this stage? | | |
| Section 2: General Understanding of Pharmacogenomics Testing | | | |
| 1. What is your understanding of PGx ? | | | |
| 1. How familiar are you with the concept of pharmacogenomics testing? | 1. Have you personally experienced a situations where PGx played a role? 2. Have you come across any real-life examples of PGx in healthcare? 3. Were you introduced to PGx during your time in a different country? | | |
| 1. In your prospect, what is the role of pharmacogenetic testing? | 1. How it could improve the medication dosing? 2. How it coud improve the medication efficacy? | | |
| 1. What information do you have regarding the implementation of PGx tests in the UAE? | 1. Did you witness any PGx implementation in the UAE? 2. Do you know the cost of pharmacogenomics test in the UAE? | | |
| Section 3: Perceptions of Pharmacogenomics Testing | | | |
| 1. Dose your company cover the PGx tests? | | | |
| 1. On what criteria will your company make decisions regarding the coverage of pharmacogenomic tests? | | | |
| 1. What are the potential benefits of integrating PGx testing into the UAE's healthcare system? | | | |
| 1. What are the potential risks of integrating PGx testing into the UAE's healthcare system? | | | |
| 1. What Concerns about the Ethical of pharmacogenomics testing could arise in the UAE? | | | |
| 1. What Concerns about Legal Aspects of pharmacogenomics testing could arise in the UAE? | | | |
| 1. What are your beliefs regarding the demand for pharmacogenomics? | | | |
| 1. How do you assess the return on investment (ROI) of incorporating pharmacogenomic (PGx) testing into healthcare practices in the UAE? | | | |
| 1. Do you think it necessary to conduct pharmacogenetic testing for everyone, or should it be limited to specific groups؟ | | | 1. Do you think it should be exclusively conducted for the companion gene test? 2. Do you believe it is appropriate for insurance companies to have the authority to restrict reimbursement for certain drugs exclusively to patients? 3. Do you think it should be limited to specific groups, considering that Medicare Advantage policy guidelines suggest coverage only when there's a potential drug-gene interaction relevant to the treatment being considered reasonable and necessary? |
| 1. If you decide to cover the PGx test do you prefer to have these specialized labs to be inside the UAE ? | | | 1. Do you think its better to do it abroad? 2. If yes what do you think about the return out if the result? |
| 1. Do you think the implementation of PGx test align with the UAE vision? | | | |
| Section 4: Financial Barriers and Cost-effectiveness | | | |
| 1. What is your opinion on the significance of pharmacoeconomic or cost-effectiveness evaluations in influencing termination and pricing decisions? | | | |
| 1. Do you think that insurance companies may use pharmacogenetic test results to deny healthcare coverage | | | |
| 1. What do you think are barriers and challenges to implementing the Pharmacogenomic test in the UAE healthcare system? | | 1. Experts attributed the most critical barriers to the future development of personalized medicine. 2. Economics and reimbursement, in particular, are the most essential obstacles 3. Lack of evidence supporting its efficiency. 4. Pharmacogenomic test cost. | |
| Section 5: Integration and Recommendations | | | |
| 1. What steps or strategies should be taken to facilitate the successful integration of pharmacogenomics testing into the UAE healthcare system? | | 1. The engagement of stakeholders, including patients, pharmacists, providers, and administrative leaders 2. The evaluation of evidence involves an internal committee with proper representation. 3. The logistics of pharmacogenetic testing, such as the role of Pathology Laboratories. 4. Integrate pharmacogenomic test results into the electronic health record. 5. The reimbursement for pharmacogenomic tests management. 6. Do you think the support of payers (insurers) to enable reimbursement of PGx tests will improve the utilization of the PGx tests? 7. evidence would be most helpful in the decision-making process for you? | |

***Table S3: TAM Constructs and Insurer Perspectives on PGx Adoption in the UAE***

| TAM Construct | Insurers’ Quotes |
| --- | --- |
| Perceived Usefulness (PU) | *“These pharmacogenomic tests are helping doctors design treatment plans, leading to more specific treatments with fewer side effects.”* (F.A.)  *“Pharmacogenomic testing may benefit patients by improving outcomes and reducing side effects, but insurance companies would not directly benefit.”* (S.A.)  *“If a patient has prescribed X medication... if having that test shows the side effect, which will make the treatment plan change, then in this case, that can support the medical necessity coverage of the test.”* (M.F.)  *“We can direct the treatment directly to this patient's proper dose and medication.”* (M.S.O.)  *“It saved money, and the cost of the treatment is shorter. I don’t want to focus only on cost-effectiveness... but if he didn’t do it, maybe it would be three years.”* (H.H.) |
| Perceived Ease of Use (PEOU) | *“Integrating pharmacogenomics into current clinical workflows is still under development here, and we need more labs offering these tests to make it cost-effective and scalable.”* (A.W.)  *“Most advanced labs and facilities are confined to tertiary care hospitals... and due to the lack of competition, there is no effective mechanism to control testing costs.”* (A.W.)  *“The test cost is significantly higher when outsourced compared to conducting it in-house.”* (M.F.)  *“If it is available here in the UAE and we have the expert people, machines…then why not here?”* (M.S.) |
| Trust in Technology | *“Strict regulations and clear guidelines can control abuse and ensure the right test is given to the right patient.”* (R.O.)  *“If utilised properly and with the right guidelines and controls in place, they would have a positive impact on the care outcomes as well as on the funding of care.”* (A.B.)  *“There are ways to control the abuse. Also, definitely there will be overutilisation, but there are ways to control that as well.”* (A.A.)  *“There would be abuse and overutilisation by the medical team... if it is not medically justified and organised by clear guidelines.”* (R.O.)  *“There will be a risk of improper use of this test for insurance companies if the facility realises that the member changed their insurance and wants to do the test again for revenue.”* (M.F.) |
| Cost–Utility Analysis | *“The one-year contract is one of the main issues... It is the insurance company's main barrier to supporting long-term secure treatment.”* (M.S.O.)  *“So I will not bear this cost if the member might not stay with me, especially with individual policies.”* (H.H.)  *“Contracts are only for one year... will it be efficient and offer savings from an insurance company perspective?”* (P.A.)  *“Regarding the financial return on investment, I don't see much visibility for the insurance companies to have this.”* (A.W.)  *“We would save additional cycles for the patient... this is how we consider the return on investment.”* (P.A.) |
